# Big Hitters: Important Factors Characterizing Team Effectiveness in Professional Cricket

**DOI:** 10.3389/fpsyg.2017.01140

**Published:** 2017-07-11

**Authors:** Leonie V. Webster, James Hardy, Lew Hardy

**Affiliations:** Institute for the Psychology of Elite Performance, School of Sport, Health, and Exercise Sciences, Bangor University Bangor, United Kingdom

**Keywords:** culture, leadership, qualitative research, sport teams, teamwork

## Abstract

While organizational psychology attests to the multidimensional nature of team effectiveness, insight regarding the most important factors contributing to the effectiveness of sports teams, especially elite teams, is lacking. An abductive method of qualitative enquiry was adopted to capture participants' construal of team effectiveness, drawing on the extant literature in both sport and organizational psychology. Semi-structured interviews were conducted with 21 players, coaches, and psychologists involved in elite cricket, with resultant data analyzed inductively initially, before being reanalyzed deductively. Although, the narratives endorsed the value of many of the deductively derived factors, other constructs more prominent in organizational psychology (e.g., trust and intra-group conflict) appeared to be *more* important than traditional sport psychology group factors. The results revealed six broad themes; culture and environment, values, communication, understanding, leadership, and unique individuals, with some gender differences apparent throughout. Based on our elite sample's construal of team effectiveness, we propose a new model representing a practical, parsimonious, and novel conceptualization of the most important attributes of team effectiveness in cricket, with conceivable transferability to other team sports.

## Introduction

Sport is littered with examples of team performances that exceed the sum of their parts (e.g., the Welsh and Icelandic soccer teams' performances in the 2016 European Championships) and such events pique the interest of practitioners and researchers alike. Although, a number of related group dynamic terms have been used synonymously within the sport and organizational literature, there is merit in distinguishing between team functioning, team performance, and team effectiveness. Team functioning refers to the relevant knowledge, skills, and abilities (KSAs) required for a group to achieve its outcomes (Rico et al., 2011). The collection of KSAs, often referred to as teamwork, operate dynamically and simultaneously (Salas et al., 2007). Despite the extant research literature having a predominant focus on teamwork, we adopt the term team functioning in the present research to more accurately account for all variables (as opposed to an exclusive focus on processes; cf. Rousseau et al., 2006) that enable a team to work together effectively. Team performance and team effectiveness are consequences of team functioning. Within professional sport the most valued consequence is the outcome of the team's performance (i.e., a win or a loss; Kozlowski et al., 2015); however, team performance metrics (e.g., win/loss ratio) fail to account for the *way* in which teams achieve their outcomes. For instance, in the sport of cricket, characterized by segregated interdependence (where members are not always required to interact with one another; Evans et al., 2012), a team can produce a winning performance due largely to the isolated actions of a single individual. Alternatively, team effectiveness refers to a more holistic perspective embodying whether a team has achieved its performance oriented outcome (i.e., a win) as well as *how* the team interacts to achieve said outcome (Salas et al., 2005). Thus, a high functioning team is likely to be highly effective. Given that this holistic perspective is rare in the sport psychology literature, the present study examined how teams interact and function in order to form a greater appreciation of what contributes to the making of effective teams in cricket.

Unsurprisingly, the study of group dynamics has been a stalwart feature of the sport psychology literature and there is an ever-growing body of research endorsing positive associations between a large number of group-related variables and team outcomes (Kleinert et al., 2012). In fact, sports research has made a notable contribution to the wider literatures on cohesion (e.g., Carron et al., 1985), team roles (e.g., Eys et al., 2006), and leadership (e.g., Chelladurai, 1990). While it would not be possible to do justice to this mass of research within any single literature review, Table 1 identifies the prominent group factors (e.g., collective efficacy, communication, roles perceptions) that have been examined within the context of sport and gives a flavor of the extent and nature of the findings reported. Although, this research provides a solid foundation for knowledge, there are some general limitations relevant for the present study. First, there has been an over reliance on the examination of interdependent sport teams (e.g., basketball, hockey) at the expense of less interdependent sport teams (e.g., baseball, cricket; Evans et al., 2012). Second, while there is some evidence that group constructs vary across context and culture (e.g., Eys et al., 2015), the use of convenience sampling from teams competing at the university level is overly dominant in comparison to professional or international sports teams. Several meta-analyses in sport attest to the overreliance on sampling from both interdependent (interactive) teams and those competing at the university level (Carron et al., 2002; Martin et al., 2009; Filho et al., 2014). Third, the vast majority of this research has investigated variables such as cohesion and leadership in isolation, thereby precluding knowledge of how the numerous factors that contribute to team effectiveness operate in concert as well as which specific factors are most influential (e.g., Filho et al., 2014). Fourth, there continues to be an overemphasis on team cohesion (McEwan and Beauchamp, 2014; Collins and Durand-Bush, 2015). Fifth, the vast majority of the research has not been explicitly contextualized within conceptual frameworks of team functioning or effectiveness (nor performance; e.g., Collins and Durand-Bush, 2015). Consequently, this large body of constructs and findings is somewhat disconnected and does not offer researchers or practitioners a clear framework for their work.

**Table 1:** Summarized literature review of factors with potential relevance for team effectiveness in cricket.

**Concept**	**Working definition**	**Theoretical references**	**Empirical support**	**Meta-analysis in sport**	**Meta-analysis in org**.	**Relationship with group outcomes**
Adaptability	How well a team can recognize a change from what was expected and alter their actions and behaviors to still achieve the same shared goals	Priest et al., 2002; Burke et al., 2006	Entin and Serfaty, 1999; Wiedow and Konradt, 2010	X	✓ (meta-analysis of team processes; LePine et al., 2008)	Positive relationship with performance. Positive relationship with communication under stress. Positive relationship with coordination.
Cohesion	The extent to which a team comes together and stays together to achieve their shared goals; The extent to which a team comes together socially, away from the sport	Carron et al., 1985; Pescosolido and Saavedra, 2012	Carron et al., 2002^*^; Beal et al., 2003^*^; Dobersek et al., 2014^*^	✓	✓	Positive, bi-directional relationship with performance. Positive relationship with viability and collective efficacy.
Communication	The verbal or non-verbal exchange of information between individuals	Sullivan and Feltz, 2003; Pentland, 2013	Mesmer-Magnus and DeChurch, 2009^*^; LeCouteur and Feo, 2011	X	Information sharing ✓	Positive correlation with cohesion. Positive relationship with performance.
Conflict	Disagreements between team members that may be accompanied by negative emotions and/or interference with the attainment of the group's goals	Jehn, 1995; Paradis et al., 2014	De Dreu, 2008^*^; de Wit et al., 2012^*^; O'Neill et al., 2013^*^; Wachsmuth et al., 2017	X	✓	Negative relationship between conflict and member satisfaction and commitment. Positive relationship between task conflict and performance under certain circumstances. Conflict management positively related to team performance.
Coordination	Organization and integration of members' actions to work toward a shared goal	Eccles, 2010; Gorman, 2014	LePine et al., 2008^*^; Bourbousson et al., 2012	X	✓ (meta-analysis of team processes; LePine et al., 2008)	Coordination mediates the relationship between TMMs and performance. Positive relationship with team member satisfaction and team performance.
Collective efficacy	The level of shared belief a team has in its collective abilities to achieve shared goals and expected levels of performance	Bandura, 1997; Fransen et al., 2014	Gully et al., 2002^*^; Stajkovic et al., 2009^*^; Fransen et al., 2015	X	✓	Positive relationship with performance. Reciprocal relationship with performance in sport. Positive relationship with cohesion and TMMs.
Leadership	The behavior of an individual when directing the activities of a team toward achieving their shared goals	Bass, 1985; Chelladurai, 1990; Zaccaro et al., 2002	Burke et al., 2006^*^; Callow et al., 2009	X	✓	Positive relationship between transformational leadership and performance, cohesion, and collective efficacy. Task and person focused behaviors both related to team effectiveness.
Goal setting and planning	The way in which a team lays out how they will achieve their shared goals	Weldon and Weingart, 1993; Marks et al., 2001	Stout et al., 1999; DeChurch and Haas, 2008	✓ (meta-analysis of team building interventions; Martin et al., 2009)	✓ (meta-analysis of team processes; LePine et al., 2008)	Positive relationship between planning and use of TMMs, which in turn improves coordinated performance. Direct positive relationship between planning and performance. Goal setting interventions positively related to range of outcomes (cohesion and performance).
Resilience	The process by which a team positively and effectively adapts to stressful and adverse events	Morgan et al., 2013; Sharma and Sharma, 2016	West et al., 2009	X	X	Team resilience positively related to team cohesion, cooperation, and trust.
Roles	The behaviors expected of an individual holding a certain position (those prescribed by the organization, and those that evolve naturally)	Kahn et al., 1964; Eys et al., 2006	Tubre and Collins, 2000^*^; Beauchamp et al., 2002	X	✓	Role ambiguity negatively related to task cohesion and performance. Role acceptance positively related to performance. Athletes who exceeded role contribution expectations reported higher perceptions of task cohesion.
Team mental models	Knowledge held by members of the team that enables them to understand the requirements of the task and therefore coordinate their actions	Cannon-Bowers et al., 1993; Eccles and Tenenbaum, 2004; Mohammed et al., 2010	DeChurch and Mesmer-Magnus, 2010^*^; Filho et al., 2015	X	✓	Positively related to team processes, motivational states, and team performance. Positively related to planning, communication and leadership. Predicts collective efficacy and perceived performance potential.

* *Denotes meta-analysis*.

In contrast, there is a plethora of models on team effectiveness and teamwork within the organizational literature that have the potential to enhance our understanding of team functioning in sport. Specifically, these models seek to identify the innumerable variables that can affect the success and viability of a team (Salas et al., 2005). In an attempt to consolidate this particular literature, Salas et al. (2005) reviewed 138 different frameworks published across two decades to propose a parsimonious set of practically relevant propositions. Leadership, mutual performance monitoring, backup behavior, adaptability, and team orientation were advanced as the core components, which are themselves transformed and facilitated by three coordinating mechanisms; shared mental models, mutual trust, and closed loop communication. These components appear in many other team effectiveness/teamwork frameworks, and Salas and colleagues make valuable suggestions as to how they can be applied to the development and maintenance of teams. However, the framework *as a whole* has yet to be empirically tested and this is a shortcoming of the organizational literature more generally with models commonly including many factors to represent the complex and multidimensional nature of team effectiveness, but with little direct empirical evidence to support them.

Encouragingly, one relatively recent addition to the sports literature warrants particular coverage. McEwan and Beauchamp (2014) proposed a model of teamwork and team effectiveness in sport that amalgamated two prominent frameworks from organizational psychology; Mathieu et al.'s (2008) Input-Mediator-Output team effectiveness framework, and Rousseau et al. (2006) teamwork behaviors framework. As a result, McEwan and Beauchamp's model conforms to the traditional perspective of a team effectiveness “throughput” model whereby mediating attributes convert inputs into outcomes. In particular, these mediators were divided into three classes which contribute to the management of team maintenance (i.e., psychological support and conflict management), the regulation of team performance (i.e., preparation, execution, evaluation, and adjustment processes), and emergent states (i.e., cohesion and collective efficacy). The range of included mediators reflected the importance of cognitive, attitudinal, motivational, and affective states in team effectiveness, as well as the more accepted teamwork processes or behaviors. In addition, McEwan and Beauchamp acknowledged the multidimensional and dynamic nature of teamwork, noting the salience of certain mediating attributes at different times in team performance cycles, thus addressing a common criticism of team effectiveness models that they ignore the temporal experience of teams (Ilgen et al., 2005). However, whilst there is some data to support the relevance of a number of the constructs presented in the sport model, many of the constructs have only been studied at the individual level with little examination at the (arguably more meaningful) group level. Furthermore, while maintaining terminology consistent with the organizational domain (cf. Rousseau et al., 2006) might reduce the risk of conceptual misunderstanding, it almost certainly hinders the accessibility of the framework to sport practitioners and coaches, and their athletes. Similarly, in a review of sports oriented team process conceptualizations, Collins and Durand-Bush (2015) noted that despite the proliferation of deductively developed frameworks, applied information was not forthcoming from the available perspectives. They surmised that there is value in “bottom-up” (inductive) approaches to developing evidence-based frameworks of team functioning. We concur that such approaches would likely yield new information of importance for teamwork interventions that move beyond team cohesion.

The present research sought to gain a greater understanding of the multidimensional nature of team functioning and insight into the most important factors for team effectiveness in professional cricket. To this end, we drew from the rich sports literature whilst addressing some of its aforementioned shortcomings, and utilized research from the organizational setting which has largely been ignored to shape our investigation. The study also responds to recent calls in the sports literature for more comprehensive theorizing with regard to team functioning, and greater collaboration between practitioners and researchers (Kleinert et al., 2012). For instance, it is evident from the existing research that there are many aspects that could be the focus of attention with regard optimal team functioning. However, without knowing which of these aspects have the greatest impact on the effectiveness of different types of teams at different times and in different circumstances, the practicality of this literature is limited.

Given that practitioners, especially in professional sport, often have to work “out of sight” of performers, supporting coaching staff, we were also interested in understanding more about the language players, coaches, and performance directors used when discussing their experience of teams. This important applied issue contributed to the comprehensive, complementary, and flexible approach we employed to gain an evidence-based understanding of which attributes characterize team effectiveness in professional cricket.

## Methods

### Philosophical orientation

In accordance with a relativist epistemology, we adopted the belief that given the dynamic nature of teams, and the number of individuals involved, “multiple realities” of the phenomenon of team functioning would exist (Sparkes and Smith, 2009). Contrary to the deductive nature of the extant literature regarding team effectiveness frameworks, we were largely interested in uncovering the meaning attributed to team-related experiences by those directly involved (Sparkes and Smith, 2014). More specifically, guided by a constructivist theoretical orientation we recognized that multiple stakeholders contribute to the development of team functioning, and that their construction of this phenomenon would be reflective of their individual roles within a team. To that end, we purposefully sought the perspectives of professional coaches, players, and psychologists on the development of high functioning cricket teams.

Owing to the underdevelopment of the existing sports literature on team effectiveness, yet the proliferation of corresponding literature within organizational psychology the present research involved a succession of inductive and deductive processes; an approach which can be described as abductive (cf. Ryba et al., 2012). The aim of the study was to understand the factors that contribute to team effectiveness for players, coaches and psychologists involved in professional cricket (inductive), whilst simultaneously establishing whether participants' experiences could be understood through a number of pertinent group-related constructs (deductive). The abductive approach enables dialectical movement between everyday meanings and theoretical explanations (Sparkes and Smith, 2014).

### Participants

After obtaining institutional ethical approval provided by Bangor University's School of Sport, Health, and Exercise Sciences ethics committee, and individual informed consent, a total of 21 individuals participated in the study. This included seven professional coaches, seven players, two managers, and five applied sport psychologists (6 females and 15 males, *M*_*age*_ = 36.05 years, *SD* = 8.67) employed by the England and Wales Cricket Board (ECB), or an English First Class County cricket organization (see Table 1 of the Supplementary Information for details).

Consistent with qualitative methodologies (Patton, 2002) and procedures adopted in related research studies (e.g., Eys et al., 2015), a purposive, criterion sampling approach dictated the recruitment of those with specific knowledge and experience of the phenomena of interest (Sparkes and Smith, 2014). The principle criterion was that coaches and players had been involved in professional First Class County cricket, or Women's County cricket for at least 10 years; in fact most were highly experienced within international cricket (*M* = 6.9, *SD* = 7.73). This ensured that participants had a wealth of experience of high performing teams to draw from through the course of the interviews. Given the relatively recent employment of sport psychology consultants within cricket, no such restrictions were applied to the psychologists but all worked with an international team (e.g., England senior team). Relevant personnel at the ECB facilitated contact with participants, with interviews then conducted in the early competitive season.

### Interview guide

Each interview began with rapport building questions to put participants at ease (e.g., “Can you tell me how you first got involved in cricket?”). Next, participants were asked about their current team in order to focus their attention on aspects of team membership and functioning (e.g.,“Tell me a little about the team that you are part of at the moment, what is going on in the team?”). The following section of the interview adopted a predominantly inductive mode of inquiry to explore specific indicators of team functioning (e.g., “Tell me about a team you have been part of which you would say best exemplified teamwork” and “What would you consider to be the most important aspects of teamwork in cricket?”). Many of the a priori variables of interest to the investigation were raised organically within this section of the interview, along with additional attributes not previously considered. Questions were followed up with elaborative probes (e.g., “What contributes to a team working well together?”) or contrast probes (e.g., “What contributes to teams not being able to work well together?”), enabling further detail and clarification of the significance of these constructs to team effectiveness and functioning (Patton, 2002).

Given the proliferation of extant literature on team-related constructs, it seemed prudent to draw upon variables that had been frequently cited within existing frameworks of team functioning (e.g., adaptability and coordination; Rousseau et al., 2006), extensively researched within sport (e.g., cohesion and roles), or deemed to be relevant and applicable to the particular sport in question (e.g. resilience; Bell et al., 2013). Thus, extensive review of both the sport *and* organizational literatures facilitated the development of a deductive analytical framework of 11 constructs of interest (see Table 1). The final section of the interview defined each deductively derived variable in turn before asking participants to comment on their experience of the variable in question (e.g., “Resilience can be defined as the process by which a team positively and effectively adapts to stressful and adverse events. Can you describe any situations where your team effectively responded to stressful or adverse events?”). The use of specific probes explored whether the construct was considered to be relevant to team functioning in cricket, and the circumstances under which it may have a positive or negative influence upon teams.

Seven pilot interviews were conducted to assess the extent to which the interview guide (see Supplementary Information) allowed participants to detail their experiences of team effectiveness and adequately capture the specific variables of interest. Minor amendments were made to some of the definitions used in the deductive section of the interview, and more specific probes were added to elicit characteristics of successful teams. Some of the pilot interviews were particularly lengthy, which highlighted the importance of using specific probes to maintain the focus of questioning.

### Data collection and analysis

All bar two interviews were conducted in person, with the remainder conducted via telephone to accommodate individuals' demanding schedules. The interviews lasted between 50 and 120 (*M* = 73.85, *SD* = 25.29) min and were audio-recorded and transcribed verbatim, yielding ~480 transcript pages (249,442 words). Interviews were transcribed upon their conclusion to enable the first author to gain familiarity with the data and keep a journal of initial observations. The journal was updated following each interview, providing a means by which to explore developing areas of interest, whilst also informing the decision of when we had reached a point of data saturation; when no new themes, findings, concepts or problems were evident in the data (Francis et al., 2010).

Analysis then proceeded in a number of distinct stages. Subsequent to reading each transcript a number of times over, a short summary of developing concepts was completed for each participant. An inductive process of thematic analysis (Braun and Clarke, 2006) was then employed to fracture the data into more manageable meaning units and subsequently identify themes. A “critical friend” (Sparkes and Smith, 2014) was employed at this juncture to challenge whether the raw themes were accurately represented by the selected meaning units. The data was then reanalyzed deductively, through directed content analysis. The goal of directed content analysis is to validate or extend a theoretical framework or theory (Hsieh and Shannon, 2005). Thus, each of the 11 deductively derived constructs of interest were adopted as coding categories, and meaning units relevant to their corresponding operational definitions were extracted from the data. This process was initially applied to those deductively-targeted questions in the latter part of the interview before reanalyzing entire transcripts with these coding categories in mind. Themes from both inductive and deductive procedures were compared and contrasted, then combined to produce meaningful groupings of the data. Finally, we conducted a secondary analysis of all themes to examine potential gender and role differences.

### Credibility and trustworthiness

It is increasingly recognized that methods or techniques alone cannot attest to the quality of qualitative research (Sparkes and Smith, 2014). Thus, rather than attempting to adhere to a particular set of criteria to develop trustworthiness and rigor, the present study was guided by elements of Tracy's (2010) “big-tent” criteria considered most fitting to the purpose of the research. The concurrent application of inductive and deductive methods contributes to the development of *rich rigor* by recognizing the scope and context of previous research literature, whilst also allowing for the identification of additional constructs of interest. This, in combination with the collection of rich and abundant data ensured that the complexity and nuances of the data were not missed. The use of data-source and analyst triangulation, alongside respondent validation, also augment the *credibility* of the research. Specifically, the collection of data from three divergent perspectives (coach, player, and psychologist), and the collaboration of all three authors to converge on the final themes and framework represents a process of triangulation which enabled different facets of team functioning to be explored. In addition participant reflections were sought on both their individual transcripts, the derived themes and final model to ensure a correspondence between researcher findings and the understandings of participants (Tracy, 2010). The result is research that *resonates* with the reader, and demonstrates *meaningful coherence* by successfully illustrating individuals' experiences of factors that contribute to team effectiveness.

## Results

Consistent themes were evident throughout the interviews that indicated the importance of several core components of team effectiveness in cricket. Although, each of the deductively derived candidate constructs were generally endorsed by the participants as having some relevance to team functioning in cricket, their narratives highlighted several components that they viewed as being *more* important than these traditional group factors. Additionally, the nature of these new components was qualitatively different to but provided an effective backdrop for acknowledged group process (e.g., coordination) and emergent states (e.g., team mental models; TMM) to develop. We present our participants' construal of team effectiveness, describing each theme and where possible, its apparent function, its development, and how it relates with other group variables. The analysis resulted in six broad themes or components: culture and environment, values, communication, understanding, leadership, and unique individuals. The components appeared to be valued similarly across participants, although some gender and positional differences were apparent.

### Culture and environment

The majority of participants referred to the importance of creating an environment that would sustain effective teamwork, “The environment for me creates where the group is going, how you want them to behave, setting boundaries” (Female coach 6- females: this denotes a female coach working in women's cricket). Although, predominantly discussed by coaches and psychologists, players also recognized the importance of having an environment in which a team could thrive, “It doesn't matter how many good players you have, if you haven't got the right team dynamic, if the environment's not right, then you're going to go astray pretty quickly” (Player 6- male). Participants saw the coach as primarily responsible for the creation of this environment (“My role as a leader is to trigger that environment and that culture and pave the way”; Male coach 5- females) whereas, the captain and senior players were tasked with policing the environment through the reinforcement of values,

You want experienced people to be able to run the team and actually enforce the culture and the values that you're trying to implement…but you need policemen, effectively, in the team that can look after those values, and protect [them] (Male coach 2- males).

Coaches and psychologists considered an effective environment to be one in which there was a relative absence of fear of judgement from the group, and individuals felt able to “be themselves, on and off the pitch. ‘I'm the cricketer I want to be, and it's OK to be me. And then off the field, I can be who I am within the context of the group”’ (Male psych 1- males).

The term *safe environment* seemed to represent a concept based on trust, and a freedom to speak openly and honestly, “Having an environment where you can have that open, clean feedback is really, really key…knowing that you're not going to be judged because of what you're going to say” (Male psych 4- males). The narratives suggested that a safe environment allowed players to operate and play with freedom, and without fear of being judged or criticized, “It's alright for me to come in and play my game” (Male psych 1- males).

Although, minimal sex differences were apparent, management staff (e.g., coaches and psychologists) were more aware than players of both the need to create an appropriate environment and what such an environment might look like. This may be because staff considered the creation of an effective environment to be principally their responsibility.

### Values

Values, as “a way of working, a way of behaving that enables us to go about our business on a daily basis consistently, to work toward our team vision” (Male coach 5- females), were seen as central to an effective team culture and environment by all participants.

[The coach] coming in and really making a big emphasis on our culture and how we can live and die by our values is something that I think has been instrumental in us being as successful as we have been in the last 18 months (Player 7- female).

The values held by teams included, “enjoyment is a value that we want to instill…honesty is another one. Trust is another one” (Male coach 2- males); “responsibility, excellence, commitment” (Player 3- female).

The adoption of a set of team values appeared to provide players (and coaches) with standards by which to hold themselves accountable. They defined what behaviors were (un)acceptable, and provided markers by which to evaluate whether team members were buying into the team vision, “Having the values as everyone agrees to them gives us something to measure ourselves against…They give you something to check yourself against and check other people against…they give the team a common focus” (Player 3- female). As alluded to earlier, coaches and psychologists generally believed the most effective way to ensure individuals were being accountable for their actions and behaviors was for players to take responsibility for policing the values. Overall, values seemed to create the most effective culture and environment; providing guidelines for the behaviors that would facilitate the development of a highly effective team. Interestingly, females spoke more of the importance of values; particularly trust and honesty.

#### Trust

Trust appeared to be an indispensable component of team effectiveness; referred to by all participants. Psychologists, in particular, spoke at length about the importance of trust, and this is reflected in the following quotations, “That trust aspect is crucial…you might not necessarily get on with people, but you trust they're actually doing what's best for the team” (Player 6- male). Trust between team members predominantly referred to the belief that individuals would commit to and work hard for the team, and that they had the ability to perform the role that was required of them by the team. Belief that a team mate was committed to the team was established through training and practice, by seeing what players were actually doing,

[Trust] comes from everything you do in practice to ensure players see what each other are doing, working incredibly hard…Rather than players questioning each other on whether they're doing their work smartly away from camps…it's trusting your teammates to be doing that work away from here (Male coach 5- females).

The second element of trust, belief in the ability of the team and others, seemed to be closely related to collective and other efficacy. In short, individuals needed to believe in team mates' ability to perform “in the middle” in order to trust them, “It's your trust in the guy to do his job…you're trusting yourself with your bowlers or batters to do their job” (Player 5- male).

As well as trust in team members, participants spoke also of the importance of players' trust in the leadership; coaches, management, and the captain. Male and female players emphasized the value of this trust, “In terms of building trust between the coaches and the players, that's really important and I think when you have that trust, you have players who really want to play for you, really want to fight for you” (Player 3- female). Ultimately, trust in the leadership resulted in individuals following the direction and example set by the leader, “[When] you know you trust the leader. I think you get followership. I think that's the bottom line” (Male psych 3- males). This led to team members accepting advice, and implementing the processes and procedures established by the leaders, “There's got to be a level of trust to say ‘right OK, that's great. Those are the plans. I'm going to try and make sure we can execute that as a team”’ (Male psych 4- males).

Participants also discussed their experiences of lack of trust; without trust between players, communication became more challenging, with feedback either not being given or interpreted in a manner that was not intended. Consequently, conflict was seen to become more likely, for instance, “you can't challenge without trust. Challenge without trust is like a war zone” (Male psych 3- males). A lack of trust in the leadership and management was also seen by psychologists and players to have particularly deleterious effects on a team; ultimately, resulting in a lack of buy-in, “The lack of trust …between the head coach and some of the senior players resulted in them not buying into the strategy, and them not ultimately performing as well as they could” (Male psych 2- males).

#### Honesty

The importance of trust within the team environment was closely linked to another core value, honesty. Interestingly, although psychologists emphasized the importance of trust, honesty was referred to more frequently by coaches and players. The two values were often cited as fundamentally important however, and influenced one another, “I think the more honesty, and the trust you have within each other can only benefit that team in good ways” (Female coach 6- females). Many participants discussed the need to build trust in order to develop and encourage honesty, as one player observed in relation to trust between players and staff, “It's important in building up a trust between people within the environment so if there is a problem…you already have that trust built up, you know you can go and speak to that person if there's a problem” (Female player 3).

Not only was trust thought to encourage honesty, participants suggested that there might be greater acceptance of honest feedback if there was trust between individuals, “If you trust your teammate is doing it [giving honest feedback] for your good, then so be it. You might not like it, it might not be what you want to hear, but ultimately it's a better environment” (Male coach 2- males).

All participants believed that the best teams consistently strived to create an environment where individuals could be honest with themselves and honest with one another. Across all narratives, participants reflected upon the importance of players giving one another honest feedback. This type of feedback enabled recognition and correction of mistakes facilitating the adaptability needed for a team collectively moving toward its shared vision (e.g., “We've got to be honest and up front with each other. It's not a personal attack, it's those 1–2 percenters that we want to get better as a team and, until you can honestly review your performance, you won't get there”; Player 7- female). Honesty was also considered necessary to challenge team members on their behaviors, “I don't think people should be too nicey nice in the dressing room. If someone isn't doing it right make sure they know it, and tell them. They can disagree …but ultimately you can sort things out” (Male coach 2- males). This form of communication served to call people up on behaviors considered to be outside of the values ascribed to by the team, or the roles and responsibilities of the individual. By monitoring the agreed values, players took responsibility for upholding the team's culture and standards.

Many participants referred to these honest challenges as “constructive conflict”; different to “destructive conflict” in that it was “in the open…It's helped by guys having a better understanding of each other and having a mutual regard for wanting the individual to improve, the team to improve” (Player 2- male). Destructive conflict, on the other hand, tended to be more personal, and lacked positive intent,

…[it's] pointing fingers and it's blaming. So it's not about me telling you this so we can get better, it's about me telling you this so you can feel worse about yourself and I can feel better about myself. For me it's just taking the team bit out of it, and it's an exercise in blame (Male psych 2- males).

Through the generation of ideas and ways to improve, constructive conflict was thought to have a positive impact upon teams. More specifically,

It's important that players get opinions out there, and actually that conflict may be a turning point for a team that either isn't performing well, or needs something to occur to create a spark in that group which either then galvanizes a group, or gets them on the same page (Male psych 5- females).

Conversely, destructive conflict appeared to have a particularly negative effect, “…we had a lot of conflict in the dressing room…guys couldn't see past their own little feud…We got relegated that year and it was just nasty” (Player 2- male).

Without honesty, teams had the potential to breed mistrust, divisions, and conflict,…

That honesty with yourself, honesty with your teammates, will make a good team. And if you don't have those features I think…it just creates obstacles. And suspicions, mistrust…[Without honesty] it becomes fragmented. People look after their own patch, and probably go for individual goals rather than team goals (Manager 1- male).

The main gender difference emerging from the narratives related to honest communication. Female Player 7 observed, “I think women are generally…not great at taking on criticism because they take it quite personally.” Equally, female Coach 6 stated that “as soon as you say ‘honest,’ or ‘I'm going to give feedback,’ the girls cringe because it's going to be something that they're not comfortable with and they don't want to hear.” Males however, were thought to be more open to challenge and criticism than females, and able to separate cricket-related feedback from something more personal (e.g., “there's a greater openness to conflict and challenge in a male population…The ability to separate it from individual or task is potentially easier, and it comes back to ‘it's cricket,’ and what's said is said and it's done, dealt with”; Male psych 5- females). Consistent with this distinction, all participants involved in women's cricket reinforced the importance of values for creating the most effective environment. Honesty, in particular, provided players with accountability through which they were impelled to be honest for the greater good of the team. With honesty as a core value it appeared more likely that confrontations would be interpreted less as a personal attack (e.g., “Now [the players] don't see [honesty] as a personal attack. Everyone is just trying to make the team better”; Player 7- female).

#### Responsibility

Having a sense of personal responsibility was also frequently cited as a core value. The creation of a responsible environment was seen to develop through individuals being honest with themselves. This required team members to have a good level of self-awareness, and the ability to reflect honestly on their own performances,

[Honesty] has helped with people really trying to take accountability for their performance…and as well as being honest with other people, be honest with themselves and really reflect on the game and think about how they've performed…do they need to improve, did they fall short? (Player 3- female).

Personal responsibility was thought to be of greatest value to a team when players openly admitted mistakes and shortcomings to the team as a whole (e.g., “It's about being honest with yourself…the team environment that produces an atmosphere that somebody can stand up and say ‘I was wrong, I'm sorry,’ not ‘it happened for this reason, it's your fault’…That is the culture you want”; Manager 1- male). Moreover, in order to create an environment that encouraged honest communication and personal responsibility, many of the participants spoke of the need for senior players and leaders to role model these behaviors and set a precedent for players to follow, “I put my hand up and exposed myself on something I'd f^*^∧ked up on previously…I'm going to try and role model that behavior of exposing myself…, with the hope and expectation that then other people would follow” (Male coach 4- females).

### Communication

Communication permeated many of the factors discussed in the narratives. In line with the value of honesty, open and honest communication, discussed at length by all participants, appeared to be a highly valued form of communication. However, female coach 7 countered this, suggesting that the use of honest communication in teams is more complex than merely being honest all of time,

I think there's got to be some constructive feedback communication in there, there's got to be communication in and around what you're feeling and what's going on, communication in regard to what are our team goals and objectives, “what do we want to get out of this?” But I just don't feel that it has to be honest all the time.

Rather than always being honest, the function of communication was to provide clarity around team relevant information in order to enable a shared understanding to develop as illustrated by male psychologist 2's sentiment,

In my mind, there's a clear strategy, and a clear goal that you're trying to achieve, or a clear way that you're trying to play, and people…know how they fit into that…when it's done well they are communicated up front, and expectations are communicated around those behaviors they're going to exhibit in those roles.

Male coach 3 stressed the importance of communication by saying that, “You can have the best framework you like, but unless [every]one knows, and it's effectively communicated and effectively reviewed regularly, then it's worthless.”

#### Ineffective communication

Conversely, ineffective communication failed to provide clarity, and resulted in a lack of understanding of important issues,

Ineffective communication would be where the players…don't know the principles of the environment, they don't know what's expected of them, they don't know what they're accountable for, they don't know what their role is. Anything which leaves them in a confused place like that I think is poor communication (Male coach 3- males).

Any ambiguity resulting from a lack of information made it less likely that individuals would follow the same (bowling) plans, resulting in inconsistent, or uncoordinated performances.

#### Destructive communication

Whereas, ineffective communication failed to provide necessary information and interfered with team understanding, destructive communication seemed to conflict with team values, was associated with a lack of trust, and resulted in greater potential for conflict. This included communication that was not open, but took place behind peoples' backs, or manifested as rumors, “As soon as you start hearing rumors of signings, or underlying currents of certain people are bringing in…conflict without realizing, you start to undermine the team dynamic” (Player 1- male). Moaning and complaining was another form of destructive communication that had the potential to disrupt the team, and reflected a level of discontent and lack of trust, “My only experience of it [a team about to fail] was senior players sniping ‘he's sh^*^t, he doesn't do this, he doesn't work hard enough, he hasn't got a clue what he's doing’. That's the sign the wheels are about to fall off” (Male coach 3- males).

### Understanding

Shared understanding across a team, established through open communication, was discussed by all participants as being particularly influential. Indeed, female player 4 thought the single most positive influence on a team was,

…learning how each other works, and learning what makes each other tick, so that when you go out there you know exactly how the girls want to play- like their strengths, and you understand that, and then you can take that together as a team and go forward with that knowledge of each other.

This theme comprised of an understanding by players of their team members' personalities, leader understanding of individuals' personalities, an understanding of players' games (i.e., individual capabilities), and a shared understanding of task-related issues or TMMs.

#### An understanding of individuals' personalities

An understanding of individuals' personalities was discussed at length, and highlighted (particularly by players) as important for knowing when and how to best support team members,

When you've learnt those different traits about each individual it helps the team…maybe someone who is going through a tough spot on the field, you can understand how to help them react and go through that, and what support is needed…So I think that's where it's very important (Player 1- male).

Understanding enabled players to approach and communicate with team members in the most effective way, where failure to do so could result in irritation or frustration,

Knowing how someone ticks off the pitch is just as important as on the pitch because if you don't understand how they like to be spoken to, you can snap at someone on the pitch. There are certain people who like direct feedback, there are some people who like to reflect a little bit more (Player 7- female).

Although, most participants stated that not all team members would necessarily get along, they acknowledged that if individuals could appreciate and understand differences, then frustration would reduce. Furthermore, many of the participants discussed the use of personality profiling as a means by which to better understand team members, and using that knowledge to appreciate individual differences. Coaches, players, and psychologists all reflected on the value of such processes, “Doing personality preferences is a big insight…it has a huge slant in terms of helping them to develop and understand, and improve their appreciation of others” (Male psych 5- females). Nevertheless, this theme may have been particularly discussed by players because they have greater first-hand experience of knowing how understanding fellow teammates can influence team effectiveness in the field. An understanding of others' personalities enabled teammates to recognize when individuals might need support, and appreciate how best to provide that.

#### Leaders' understanding of individuals' personalities

It was considered to be particularly important that both coaches and captains, as leaders of the team, developed an understanding of individual personalities. Many participants felt that this knowledge contributed to leaders being able to get the best out of individuals, and ultimately the team. This was a view shared by coaches, players, and psychologists across male and female teams. Male player 1 suggested,

[The coach] understands the individual dynamic in the changing room, what works for individuals. And the quicker he picks that up when he comes in, the better that team is going to function…There's so many individuals that make up a team. Different kind of individuals, and if the coach realizes that quickly he'll get a good team out of it.

#### Understanding individuals' capabilities

Beyond the understanding of personalities, participants also spoke about understanding the way in which team members played. Time spent training together gaining this knowledge improved coordination (e.g., “Understanding other people's games is quite important. So the more teams spend time together, the more they can second guess what somebody is going to do, which helps”; Male coach 1- males). Furthermore, female player 4 felt this could make the difference between winning and losing,

Having trained with all of the girls over the summer I've got a massive increased awareness of what people can do…the girl that I bat with at [county], I know that if a ball is bowled in a certain area, she's going to hit it in that area, and I've already started running. Little things like that might only make 0.5% of differences, but you add them all up and actually it can be the difference between winning and losing.

#### Team mental models

The understanding of elements pertaining to the team's task that was discussed by participants relates to the concept of TMMs (Klimoski and Mohammed, 1994) representing shared knowledge about key features of the team's environment. Across the participants (particularly the psychologists) this knowledge was seen to develop from clarity around a team's vision, goal, plans, and individual roles,

If you're chasing 300, “how are we going to go about it? Does everyone understand it? Does everyone see why that's the route we're going to take?”…I'd say possibly more than any other sport I can think of, [cricket] is about that tactical collective mindset (Female manager 2- females).

Male psychologist 5 reflected on his experience working with a female team, “[TMMs have] been really influential in the players in the team having consistent success…I think by having a framework in which the players went out to play a brand of cricket it gave clear direction to how the players were going to do it.”

Clarity of individual roles, in particular, was considered to characterize effectiveness when shared across the team; communicated clearly to the individual who occupied the role and to the team as a whole. This enabled a shared understanding which resulted in less blame and more acceptance if things went wrong, and was spoken about extensively by all participants,

It's really important to the group that they know what each individual is trying to do. So if this guy is a wicket taker and he might go for a few runs, if the rest of the team are going “oh f^*^∧king hell, why's he trying that?,” whereas if they know, they're likely to go “yeah come on, keep going, you're going to get a wicket” (Male coach 3- males).

Understanding one another's roles ensured that players were less likely to place blame on other team members. This increased the acceptance of errors/mistakes, and so reduced the likelihood of conflict. Thus, it appeared to be a highly-valued quality within all narratives. In addition, understanding task-related elements enabled the team to work together and coordinate effectively.

If we know we're trying to bowl leg-stump Yorkers then if you're fielding at square leg you know where the ball is likely to be going…there's definitely a shared understanding of that. Same with batters that you clearly understand this is this guy's strengths, and this is his go to shot to get off strike, and if he hits it there…I've got to run my first 2 as hard as I can (Male psych 2- males).

As female coach 6 explained, the understanding required for coordinated action came down to the team's preparation, and communicating the necessary information to the team,

It's important for the bowler to understand where they want to bowl, and then it's important for the fielders to understand where they're trying to bowl to where they stand…it comes back to the role and the clarity in your game plan and everyone's clear on what you want to execute.

In contrast if a team's game plans and roles were not clearly understood by team members, then participants discussed the potential for this to interfere with coordinated performance. Ultimately this was perceived to result in individuals striving toward individual rather than team goals (e.g., “If you don't have it [understanding] then you get some cracks appearing because people start questioning things, and if you don't have a coherent unit, you've got that individual element”; Player 2- male).

### Leadership

Leadership appeared to play an important role in many of the teamwork variables discussed, “My experience would be the leadership…when that was done best, that was best team functioning, or team environment that was created” (Male psych 2- males). Moreover, effective leadership was considered by coaches and psychologists to be critical for a team to enjoy long term success,

You can have a group of players who are outstanding players, they're able to go out there and perform despite a certain environment and certain individuals. But to be successful for a long period of time, I think you need good leadership (Male coach 5- females).

The importance of leadership to many participants was in the creation and reinforcement of the aforementioned environment. The coach was considered responsible for creating the most appropriate environment, with the captain responsible for role modeling desired behaviors, “My role as a leader is to trigger that environment and that culture and pave the way” (Male coach 5- males). Through the creation of an environment and culture with clear vision and values, leadership appeared to provide the team with direction (e.g., “I think you get good leadership, I think you get direction”; Male psych 3- males). This direction came from leaders providing a clear inspirational vision and leading by example.

#### Inspirational vision

Many of the participants referred to effective leaders as being inspirational and passionate (e.g., “He [the captain] was ridiculously inspirational…he was very good as a leader”; Player 1- male) and this, in turn, transferred on to others,

You can see that [the captain] just absolutely loves the game, and wants everybody to be better, and just wants to win. There's that desire for success, and then obviously because she is so determined and focused, you want to play for her, you want to do well because she's your captain (Player 4- female).

#### Lead by example

Another characteristic of effective leaders evident in the narratives was that they led by example. This was considered to be particularly important for role modeling the behaviors required by the team's culture and values. In this respect, the leader was responsible for setting a precedent for others to follow,

If you're leading as a captain, you have to set by example…You do have to be the one that this is an example of the team that we're going to be. These are the characteristics that we're going to portray, and this is how we're going to be as a group of individuals (Male psych 4- males).

#### Role of the coach versus captain

Nearly all coaches and players believed the coach and captain occupied different, but complementary, leadership roles. As alluded to above, effective coach leadership involved the implementation of values, roles, and games plans, whereas captains inspired their teams to move in a single direction, setting an example for others to follow. This complementarity was also discussed across team settings with the coach responsible for the environment, training and practices, and the captain predominantly responsible for leadership on match days,

The coach—very much in charge of the overall set-up…But there was a kind of ceremonial hand-over from the coach to the captain prior to the game starting and then, while the game was on, anything that goes on the field is the captain's stuff…I suppose in that respect, each of the leaders appreciating each other's role and when whose time it was to do what (Player 6- male).

Leadership seemed to be most effective when both parties understood and accepted their respective roles and responsibilities, as female coach 6 suggested, “The best working relationships I've had and for teams have been the coach and the captain are really clear on which bit is theirs and which bit is the captain's.”

#### Coach-captain relationship

Despite differing leadership responsibilities, all participants emphasized the importance of the coach and captain having a good relationship, and being able to present a united, cohesive leadership approach to the team,

The captain-coach relationship is so, so, important. Having similar philosophies on the type of cricket you want to play, on the game generally, on the type of people you want…if you start getting conflicts there, then it becomes really difficult very quickly Player 6- Male).

Conversely, any divisions between the coach and captain had a negative influence on the team, as male psychologist 5 reflected of his experience with a female team,

Initially [coach] and [captain] didn't have a very strong relationship, and they had very different opinions on where the team was going and what was success. And they had very different opinions on how to go about those plans, which in the short term created some tension and that had a big impact on the culture, the confidence, communication and performance. When those two became more aligned…got some clarity on where they, as a unit, wanted to go, and what they wanted to achieve and how they were going to achieve that…I think that created the environment which enabled the processes that needed to be in place…which then had a knock on effect because there was consistency, and everything aligned toward one goal.

The benefit of a strong coach-captain relationship appeared to be a consistent and unified approach to leadership; one which gave players a clear direction whereas, a lack of unity between the coach and captain could create ambiguity and confusion.

#### Ineffective leadership

Given the central nature of leadership for teams it should come as little surprise that several participants referred to a lack of leadership, or ineffective leadership as being one of the most detrimental influences on a team. Essentially, a lack of leadership failed to provide a team with any direction (“We were lacking any kind of direction, any real enthusiasm from [the coach], and we had a fairly poor captain who disappeared off the face of the earth halfway through the season. So I think however strong a team is, you'll struggle”; Female player 3).

### Unique individuals

A large proportion of participant narratives referred to the components that contributed to team effectiveness. However, the influence of selfish or individually-oriented players on a team was also discussed by all participants as having the greatest potential to disrupt a team. Despite the fact that all participants considered an individual becoming more important than the team as having the single most negative effect upon a team, selfishness represented an interesting paradox, given that “the nature of cricket is that it's an individual sport played by a team” (Male coach 3- males), and that when batting or bowling players have primarily individual roles to fulfill. Male manager 1 commented that, “You could say to a batsman that ‘I want you to be really selfish and don't give your wicket away?’ And then ‘I want you to play for the team.’ Those are complete opposite statements.” Unique individuals were such because of their unique but contrasting contributions through skillful performances whilst also being capable of disrupting the team. For instance, in certain contexts unique individuals contributed positively to a team, as they were particularly talented,

…you've got some very good players, some individuals that may well be selfish inside, but actually the selfishness makes them better players…someone can just love batting all day and actually batting all day wins us the game because they get lots of runs, but I think when it comes [at a] detriment [to] your teammate, that becomes an issue (Male coach 4- females).

Nonetheless, there appeared to be a point at which unique individuals became too destructive, as a team was considered to be at its best when all members bought in to the collective goals and ambitions. Participants explained that unique individuals can disrupt team functioning when the individual becomes more driven by their own agenda than that of the team (e.g., “When people have their own agendas it makes it quite tough…if their agenda is to do stuff that is against what the team is trying to do, then that obviously then has a detrimental effect on team performance”; Male coach 2- males). Moreover, most participants shared the view that once an individual's agenda started to interfere with what the team was trying to achieve, then that individual should be omitted from the team. There was seemingly a tipping point, beyond which the positive influence of the individual's performances was outweighed by their negative influence on the team,

It doesn't really matter if you're a fantastically talented individual, and you score 100s of runs, but you are detrimental to the rest of the team, and you bring a lot of the team down with you…you're not worth having around…There's a bit of a cost-benefit analysis there (Male psych 4- males).

Coaches, psychologists, and players all discussed the paradox of unique individuals, with consensus that there comes a time when they become too disruptive. However, a gender difference was evident concerning unique individuals as there seemed to be limited experience of these individuals within female cricket teams.

### Other indicators of team effectiveness

Finally, there were a number of factors that appeared to represent visible indicators of an effective team. One of these was the extent to which the team remained physically united both on and off the field, evident in one coach's reflection of an ineffective team, “There was never any teamwork. They batted and disappeared. There was no sitting round together” (Female coach 9- females). This physical togetherness was also often evident in a team's celebration of one another's successes; frequently referred to as a marker of an effective team, “You can tell how much a team are a team in the way they celebrate a wicket. They're genuinely pleased for their teammates” (Manager 1- males). Another indication of effective team functioning was apparent from a team's behavior in the field, “The moment you watch a team fielding…you'll tell a lot where they are as a team. If they're all together…and [all] helping each other out you'd probably say the team is in a decent place” (Male coach 2- males). A final marker of effectiveness referred to was a lack of scepticism within a team, “Scepticism not being around, and having collectively open minds. That was probably more powerful for the improvement of the team and its winning” (Male psych 1- males).

## Discussion

The present research sought to gain a greater understanding of the multidimensional nature of team functioning and insight into the most important factors for team effectiveness in professional cricket. Our method of enquiry enabled us to verify the relevance of a range of group factors with strong empirical and theoretical ties to team effectiveness, whilst also allowing participants to raise previously unrecognized constructs pertinent to their own experiences (e.g., trust and intra-group conflict). Many of the factors commonly viewed as being salient to team effectiveness (e.g., cohesion and collective efficacy) did not appear in our analysis. This is likely a result of the parallel method of enquiry used, our emphasis on participants' views concerning the most important constructs for team functioning, and sampling from professional and international sport.

**Figure 1:**
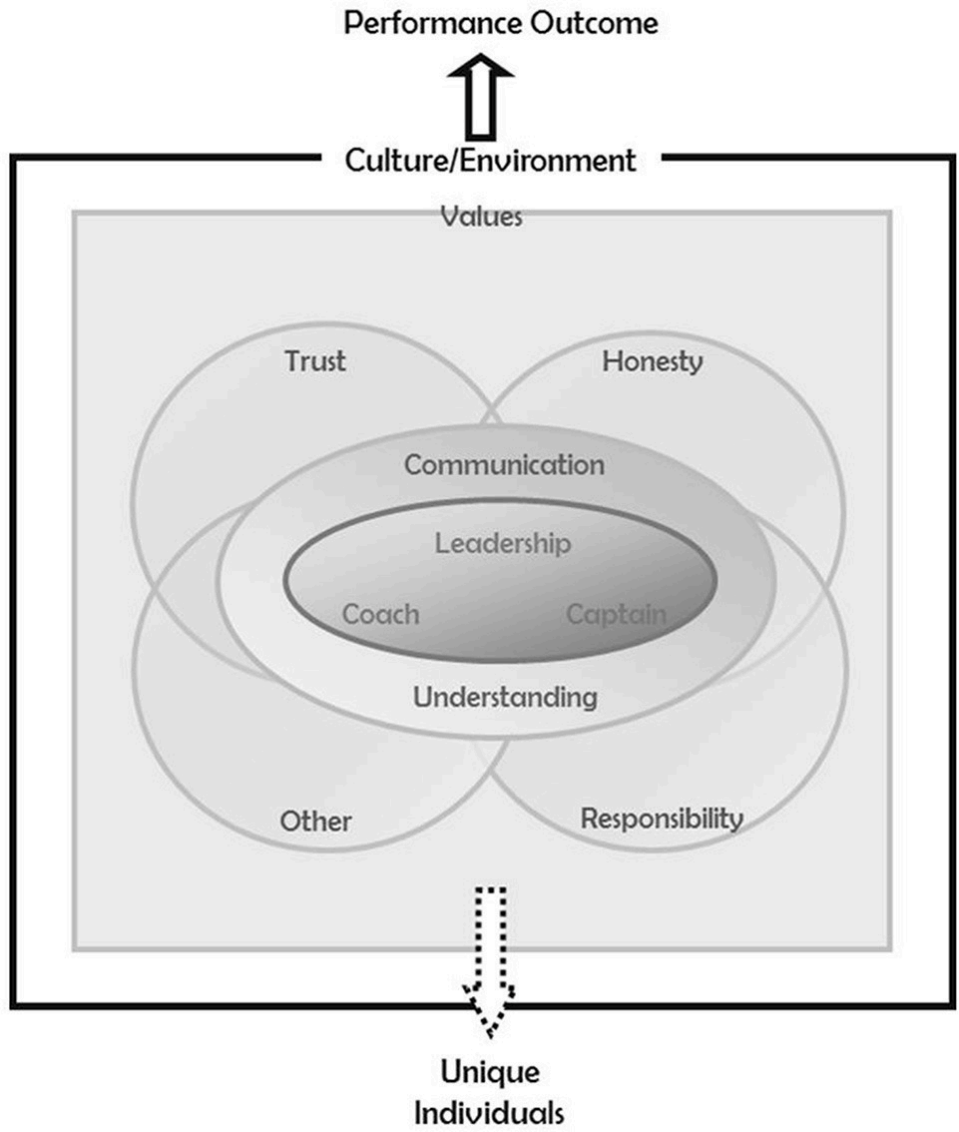
Applied heuristic of the essential ingredients of team effectiveness in cricket.

Timmermans and Tavory (2012) suggest that the aim of an abductive approach is theory construction where the researcher is led away from old to new theoretical insights. Therefore, Figure 1 depicts a novel conceptualization of team effectiveness in cricket, derived from participant narratives, that differs from existing frameworks and the models discussed in our introduction. This is perhaps to be expected given that this study is the first to use a “bottom-up” approach to develop an evidence-based framework of team functioning in sport. Our model is also representative of the language of coaches, players, and practitioners involved in elite cricket. It has a greater emphasis on broader components as compared to the more specific mediators that have been the dominant focus of previous research (cf. McEwan and Beauchamp, 2014). In fact, the discourse captured concerning the most important aspects of team effectiveness points to a number of original components that reflect more fundamental aspects underpinning the conventional group constructs reported in the literature to date; for example, culture/environment and trust. The framework in Figure 1 is a heuristic model consolidating our participants' expert views regarding the essential ingredients for team effectiveness in professional cricket.

### Summary of the essential team ingredients in professional cricket

Within sport, leadership is one of the most heavily researched constructs in group dynamics (Kleinert et al., 2012), and meta-analytic organizational evidence is supportive of a positive relationship between leadership and team performance outcomes (Burke et al., 2006). Morgan et al. (2013) contend that leadership processes enable teams to survive and thrive over time. It is unsurprising therefore, that the importance of leadership was reflected in participants' narratives, and is represented by its centrality in Figure 1. Leadership permeated all aspects included in the model. Consistent with current understanding of transformational leadership (e.g., Callow et al., 2009), and more recent research on inspiration (e.g. Figgins et al., 2016), the best leaders were seen to display appropriate role modeling by setting a positive example as well as providing a clear inspirational vision and direction for the team through a united coach-captain relationship. Indeed, Figgins et al. (2016) suggested that inspiration from leaders may enhance group functioning through increased team-bonds and identification. Although, the coach and captain occupied divergent leadership roles, presenting a united leadership approach enabled consistency and clarity of messages, and promoted trust. These findings are concordant with research that has explored the role of a sports captain in more detail (Cotterill and Cheetham, 2017). Notably, the captain's role was seen to include motivation (i.e., inspirational), embodiment of the culture (i.e., lead by example), and communications with coach (i.e., coach-captain relationship).

Working outwards, the model reflects the reported influence of leadership on the communication and subsequent level of understanding within a team (i.e., through inspirational vision, and an understanding of team members). Through open communication of team goals, game plans, and roles, a shared understanding developed. This finding is in line with the concept of TMMs as shared knowledge of key features of a teams' environment (Klimoski and Mohammed, 1994). This understanding allowed individuals to better coordinate with one another, and where there was shared understanding of individual roles, it circumvented potential conflict and frustration. Meta-analytic findings also support such a cognitive foundation to teamwork, revealing positive relationships between TMMs and group behavioral processes, team motivational states, and work related performance (DeChurch and Mesmer-Magnus, 2010). Moreover, Morgan and colleagues argue that resilient teams are able to draw on role systems and TMMs to enable a team to organize and adapt in adverse situations (Morgan et al., 2013, 2015). The relevance of team cognition has not been overlooked by sport researchers, as there is evidence of the presence of TMMs in sport (e.g., Filho et al., 2015).

Mathieu et al. (2000) delineated two types of mental models: task-related models as discussed above, pertaining to materials needed for the task (e.g., games plans, roles, etc.), and team-related models which contain expected behaviors of team members. Indeed, what appeared to be of greater value than task-related elements within the present research was an understanding of team members. Understanding others led to the provision of support to team members when required, and assuaged frustration that might develop from personality differences. Beauchamp et al. (2008) provide evidence for the importance of understanding team members in sport, utilizing a personality-preference based intervention to improve team trust and cohesion. Furthermore, our finding has similarities with the concept of emotional intelligence (Meyer and Fletcher, 2007). Indeed, the emotional awareness of individuals within high performance sports organizations influence their ability to develop and maintain effective relationships (Wagstaff et al., 2012). Unsurprisingly, a greater understanding of the emotions, personalities and behaviors of oneself and others appears to be beneficial to social relations and interactions within the context of a team. This “social capital” has been found to be a critical characteristic of teams able to withstand a range of collective stressors (Morgan et al., 2013).

The development and maintenance of a set of values; principles that drove the behaviors required to achieve the team vision, were seen as central to enabling efficient communication, developing shared understanding, and creating an effective team culture. There are a multitude of values that teams might ascribe to, but the relative importance of any value is specific to a given team and determined by the culture and vision of that team (Shoenfelt, 2011). The most commonly cited values of trust, honesty, and responsibility ensured effective interpersonal exchanges between team members, open and challenging communication, and an acceptance of mistakes. In related research with resilient sports teams, Morgan et al. (2013) found the development of a shared vision led to the ability to challenge one another against agreed standards.

Trust, in particular, was a value that participants viewed as fundamental to long-term success; referring to both a belief in team mates' abilities and trust in their intention to act in the best interests of the team. The importance of trust in establishing a high-performance culture was also apparent in a recent case study of mental toughness in an Australian Football League team. Coulter et al. (2016) found that being trusted held cultural significance in the club, and was the basis from which other values (e.g., *team first*) were developed. A recent meta-analysis in organizational psychology revealed a strong, positive relationship between both intra-team trust and team trust in the leader, and team performance (De Jong et al., 2016a). However, despite being one of the most frequently studied constructs in organizational research (De Jong et al., 2016b), trust has received little research attention in sport. The present findings, aligned with a wealth of literature in organizational psychology, suggest that future research in this area is warranted.

Constructive feedback, discussed as part of team honesty, is an important adjustment behavior that enables teams to make changes to improve performances and ensure goal attainment (Rousseau et al., 2006). It has been suggested that resilient teams gain strength through others' feedback following disappointments (Morgan et al., 2013, 2015). However, gender differences evident in the present research suggest that male teams may be more open to this challenge and criticism. Interestingly, a recent review of conflict in sport suggested that male athletes appeared to engage in more conflict behaviors and communication with their coaches than female athletes (Wachsmuth et al., 2017). Although, there is scant research regarding psychological differences between male and female sports teams (Cronin et al., 2015), Eys et al. (2015) found males to be more open with one another than females, particularly in relation to expressing and resolving conflicts. Taken together, these results point toward an underlying difference in the interdependence of males and females that requires further research.

Culture has previously been defined as a collection of shared values, beliefs, expectations, and practices across members of a defined group (Cruickshank and Collins, 2012). Within the present research an effective team appeared to develop from the culture and environment within which it was situated. Principally established by the coach, and role modeled and reinforced by the captain, high performance environments contain a clear vision of success, and a set of values. This is in line with a growing body of research which recognizes the critical influence of organizational culture and high performance environments in professional sport (e.g., Fletcher and Wagstaff, 2009), highlighting in particular the importance of leadership in the creation and regulation of such high-performance cultures (Cruickshank et al., 2015). From the perspective of coaches and practitioners, the most effective environment was a “safe environment” where individuals felt able to “be themselves, on and off the pitch.” Although, the term “safe” represents a slightly clichéd perspective of an environment that is inherently pressurized and challenging (e.g., Mellalieu et al., 2009), this term most accurately reflected participants' narratives. The construct bears semblance to the concept of team psychological safety; trust that the team will not embarrass, reject, or punish someone for speaking up (Edmondson and Lei, 2014), where the absence of threat from inside the group enables a climate in which members are comfortable being themselves. This facilitates team learning, and in turn effectiveness (see Edmondson and Lei for a review).

Overall, team effectiveness appeared to be the result of a group of individuals striving in the same direction toward a shared vision and performance outcome. This was made possible by leaders affording clarity of vision, values, and roles through open communication, and the provision of an inspirational role model for the team to follow. The influence of unique individuals within a team, at times, facilitated this endeavor through strong individual performances. However, when the individual's agenda conflicted with the team's direction, then his/her influence was considered too detrimental to the team environment. Indeed, sometimes “a single, toxic team member may be the catalyst for group-level dysfunction” (Felps et al., 2006, p. 176). Cope et al. (2010) found the informal “cancer role”—individuals who display negative interpersonal behaviors despite often being highly talented—to have a particularly deleterious effect on group functioning, with our data suggesting that male teams are more prone to this than female teams. Such players can create a dilemma whereby coaches have to decide whether the talent of the individual outweighs their potential to disrupt the team. However, it could be argued that a greater understanding of individual personalities may lessen potential frustrations, and provide leaders with information to utilize in the effective management of such individuals. Indeed Arthur et al. (2011) findings reinforce the importance of coaches knowing their athletes and personality traits such as, narcissism, when they are concerned with greater productivity.

### Limitations and considerations for future research

The present findings ought to be interpreted in the context of certain boundaries. Although, the number and variety of participants interviewed is a strength, facilitating an in-depth examination of team functioning within elite sport, the exclusive focus of the study on cricket potentially limits the transferability of the findings. Thus, a worthwhile extension of the research would be to assess whether the importance of these attributes holds true across a variety of team sports. In addition, investigating a complex social phenomenon such as teamwork through interviews alone is arguably reductionist; failing to offer a complete account of the lived experience of team effectiveness (Smith and Sparkes, 2008). Capturing *in situ* observations of critical incidents of teamwork would have minimized the constraint of relying on participant recall alone, and might have resulted in an even more comprehensive conceptualization of the phenomena. At the very least it could present an important complementary view of effective teamwork. Nevertheless, the present study clearly highlights the need for researchers to consider constructs outside of the traditional sports team literature if they are to better understand team functioning.

A logical extension to the present research would be to test whether the components included in the model can successfully discriminate between effective and ineffective teams. Such knowledge would not only attest to the import of certain attributes over others, it would also highlight areas for further investigation. Moreover, such quantitative data could enable the development of a diagnostic tool to assess whether teams are showing signs of sub-optimal functioning (cf. McEwan and Beauchamp, 2014).

Within the presented model, the importance of culture serves as a reminder that teams do not exist within a vacuum, and that there are numerous climatic and cultural factors associated with the optimal development of high performance teams (Fletcher and Wagstaff, 2009). A potential shortcoming of the present research is that it failed to explore the impact of wider support teams on effective team functioning (e.g., Collins et al., 1999), or the ways in which the constructs raised in the narratives related to the wider support team (i.e., trust). Drawing on the emerging body of research on sports oriented organizational psychology, this would be a note-worthy extension to the present research.

### Applied implications

The proposed model, formulated from the accounts of players, coaches, and practitioners involved in professional cricket provides a unique understanding of team effectiveness that is parsimonious, sport specific, and practical. Additionally, the presence of some sex differences flag more nuanced aspects of team functioning which might more effectively shape the style of communication fostered in male and female teams. For instance, it may be advisable for those involved in female team sports to be mindful of the potential impact of conflict on relationships, and ensure that such exchanges are managed in a timely manner. By retaining the terminology utilized by participants, individuals working with sports teams can draw upon this model as a guide by which to prioritize their long-term team building efforts and share ideas with coaches and players unambiguously (cf. Paradis and Martin, 2012). Meta-analytic evidence provides support for the efficacy of team training interventions, particularly workshops, simulation training, and review-type activities (McEwan et al., 2017). It is reasonable to believe, that a team training intervention targeting elements of our model might be particularly efficacious. A specific example of this might involve the structuring of debriefs (cf. Tannenbaum and Cerasoli, 2013) around the components of the model to provide teams with a clearer indication of areas for improvement, and present potential warning signs that might flag latent problems in team functioning that are about to surface. Furthermore, it is possible that the model will provide direction for coaches tasked with bringing together collections of individuals into scratch teams (e.g., for international competitions) who are expected to bond and produce results quickly. Practitioners utilizing the model must, however, be mindful of its specificity to cricket, and consider the relevance and applicability to their own team sport.

## Conclusion

There are a multitude of concepts that have been associated with group-level outcomes in both sport and organizations. Consequently, it is doubtful that a single model of team effectiveness can accurately capture all these features in detail. Furthermore, those factors of greatest importance may differ across sports, genders, competitive levels, and stages of development. Nonetheless, as a result of our thorough analytic approach (e.g., supplementary analyses concerning sex differences), the subsequent and new conceptualization offers a practical and relatively parsimonious model of team effectiveness, representative of experiences in elite cricket, which can be applied and/or adapted by those responsible for the creation of high performing sports teams.

## Author contributions

LW: Contributed to the conception of the research and study design, was solely responsible for the collection of the data, completed the transcription and analysis of all data, wrote several drafts of the manuscript in full, and had final approval of the version to be published. JH and LH: Substantial contribution to the conception of the research and study design, served as a critical friend in all stages of the analysis of data, read and edited all drafts of the manuscript, and had final approval of the version to be published. JH: rewriting large sections. This is the first study in a Ph.D. undertaken by LW and supervised by JH and LH. All authors agree to be accountable for the content of the work.

### Conflict of interest statement

The authors declare that the research was conducted in the absence of any commercial or financial relationships that could be construed as a potential conflict of interest.
