# Supplementary material for: Big Hitters: Important Factors Characterizing Team Effectiveness in Professional Cricket
Source: Front Psychol. 2017 Jul 11;8:1140. doi: 10.3389/fpsyg.2017.01140 (PMC5504155; doi:10.3389/fpsyg.2017.01140)
Supplement: Supplementary file 1 [file DataSheet1.pdf]

Supplementary information

A. Table detailing characteristics of all interview participants

Role	Gender of participant	Gender of team	Years involved in professional cricket
Psychologist 1	Male	Male	8
Psychologist 2	Male	Male	5
Psychologist 3	Male	Male	11
Psychologist 4	Male	Male	2
Psychologist 5	Male	Female	5
Coach 1	Male	Male	39
Coach 2	Male	Male	23
Coach 3	Male	Male	16
Coach 4	Male	Female	25
Coach 5	Male	Female	30
Coach 6	Female	Female	31
Coach 7	Female	Female	17
Manager 1	Male	Male	43
Manager 2	Female	Female	20
Player 1	Male	Male	10
Player 2	Male	Male	15
Player 3	Female	Female	14
Player 4	Female	Female	12
Player 5	Male	Male	17
Player 6	Male	Male	17
Player 7	Female	Female	25

B. Interview Guide

Understanding the most important aspects of teamwork in cricket.

WELCOME:

Thank you for agreeing to participate in this research. You are making an invaluable contribution to research that will hopefully inform team practices in cricket and contribute towards further research that will look to improve overall team performance.

The interview should last around an hour and a half and will be audio recorded and later transcribed. You will, at some stage, receive a copy of this back in order to confirm that the transcription is an accurate account of what you said. Are you happy to do so? I want to assure you of complete confidentiality, and all of your responses in any subsequent write-up shall remain entirely anonymous. Please feel free to decline answering any question, and you are free to end the interview at any point.

As you may have read in the overview of the research, this study seeks to find out more about some of the characteristics of cricket teams that enable them to perform at their best. As such I'd like to find out more about your own experiences of teamwork in cricket, and maybe particular things that have stood out for you from teams that you have been involved with in the past. I encourage you to draw from all of the previous teams you have been part of, not just your most recent, or that of the highest representative level. Everyone will have very different experiences of team membership and so there are no right or wrong answers. I want to know about your own experiences and views in your own words.

There are several key areas I wish to explore with you through the course of the interview. Some may come up in the discussion of your experiences, and I will weave in questions relating to those where appropriate, and those that are not raised will be discussed at the end of the interview. There is research literature about teamwork that is not necessarily related to cricket, but may be of relevance. I have a checklist of these things that I will refer back to through the course of the interview just to make sure I've covered them. Some of these you may cover yourself so I will just weave in questions of relevance as you do. To conclude the interview I will ask you to consider which of the qualities discussed are most important to teamwork in cricket, so I will be taking a note of the key areas raised throughout. This will help me keep a record and also serve as a reminder for you. Please don't let this distract you.

You are a source of great knowledge and experience in this area, so your honesty and accuracy is of great value to this research.

1. Introduction/background questions

   i. How did you get involved in cricket?
      – Number of teams (player/coach, levels, qualifications)

2. Indicators of teamwork

   i. Tell me a little about the team that you are part of at the moment, what is going on in the team?
      – Is teamwork important in the group?
      – How, if at all, is teamwork important in professional cricket?
         ▪ In relation to fielding/batting/bowling and training/touring (does it become more/less important in certain contexts?)
         ▪ When does it become less/more important?
         ▪ Is this view shared/to what extent do you think players view teamwork as important?

   ii. What is your experience of teamwork in professional cricket?
      a. Tell me about the first professional team you were part of
      b. Tell me about a team you have been part of which you would say best exemplified teamwork
         – What contributes to a team working well together?
         – What characteristics would indicate to you that a cricket team is working effectively as a unit?
      c. Tell me about the least successful team you have been part of
         – What contributes to teams not being able to work well together?

   iii. What would you consider to be the most important aspects (or one most important aspect) of teamwork in cricket?

   iv. What would a team failing, or about to fail look like? What might indicate that a team is going from good to bad?

3. Other variables
   i. There are a few other aspects of teamwork from research literature that have not necessarily been considered in sport.
      – Describe a situation in which this quality has been present

– To what extent do you think this quality is important to the performance of a cricket team?

THEMES:

1. *Cohesion:* The extent to which a team comes together and stays together to achieve their shared goals; The extent to which a team comes together socially, away from the sport
   - *Do you think both are equally important in professional cricket?*

2. *Efficacy:* The level of shared belief a team has in its collective abilities to achieve shared goals and expected levels of performance.
   - *Can you describe a team you have been part of that has had a high level of collective confidence? How influential is this is cricket?*

3. *Leadership:* The behaviour of an individual when directing the activities of a team towards achieving their shared goals
   - *Can you tell me about the most influential leader you have had?*
   - *Can you give an example of when you have been a leader?*

4. *Communication:* The verbal or non-verbal exchange of information between individuals
   - *Can you describe instances of effective and ineffective communication? What effect do these have upon the team?*

5. *Team mental models:* Knowledge held by members of the team that enables them to understand the requirements of the task and therefore coordinate their actions
   - *Can you please describe situations where team members have to understand what each other are going to do so they can effectively coordinate their actions?*

6. *Coordination: O*rganisation and integration of members' actions to work toward a shared goal
   - *Can you explain the circumstances that might require coordination in cricket?*

7. *Adaptability:* How well a team can recognise a change from what was expected and alter their actions and behaviours to still achieve the same shared goals
   - *Can you describe any situations where your team would need to recognise a change and alter their actions to achieve success? How important was this quality?*

8. *Resilience:* The process by which a team positively and effectively adapts to stressful and adverse events
   - *Can you describe any situations where your team effectively responded to stressful or adverse events? How did this influence performance?*

9. *Conflict:* Disagreements between team members that may be accompanied by negative emotions and/or interference with the attainment of the group's goals
   - *What is the content of arguments/disagreements? Effect of different types?*
   - *How is conflict managed? Examples on and off the pitch.*

10. *Planning:* The way in which a team lays out how they will achieve their shared goals
    - *Can you explain your experience of setting goals as a team?*

11. *Roles:* The behaviours expected of an individual holding a certain position (those prescribed by the organisation, and those that evolve naturally)
    - *What kind of roles are players required to fulfil in a cricket team? What are the consequences of individual not understanding/accepting a role?*

PROMPTS:

Example

a. Can you provide me with some 'for instances' of when that has been important?
b. What do you mean by that?
c. Can you provide a particular example of a team with this quality?
d. Describe what a team with good/bad _______ looks like?

Outcomes

a. What are the outcomes for a team that has this quality?
b. What are the potential outcomes if a team does not have this quality? How effective can a team be without it?
c. To what extent does this aspect of teamwork affect performance?
d. How does the presence of this quality improve team functioning or the way a team performs as a whole unit?
e. Are there any circumstances where you would not want a team to have this characteristic?

Importance in different contexts

- To what extent is this quality present in training/matches, on tour, on field/off, vs poor/good opposition, or within different sub-teams (bowlers/batsmen)?
- How important is the quality in training/matches/off the field/on tour? When does it become more or less important?
- How does being under significant stress or pressure affect this quality within a team?

CONCLUSION:

Are there any other aspects of teamwork in cricket that we haven’t covered yet that you feel have an important influence the performance of teams?

Or is there anything else you want to raise?

What one thing has the single most negative effect on a team?

What one thing has the single most positive effect on a team?

Of all the characteristics that have been considered, can you describe whether the team directs any effort towards improving any of these qualities?
